# Secondhand Smoke Exposure, Restless Sleep, and Sleep Duration in Adolescents

**DOI:** 10.1155/2014/374732

**Published:** 2014-04-07

**Authors:** Jennifer Schwartz, Joan L. Bottorff, Chris G. Richardson

**Affiliations:** ^1^School of Population & Public Health, University of British Columbia, 2206 East Mall, Vancouver, BC, Canada V6T 1Z3; ^2^School of Nursing, University of British Columbia, 3333 University Way, Kelowna, BC, Canada V6T 1Z3

## Abstract

*Purpose*. To examine whether secondhand smoke (SHS) exposure is associated with restless sleep and/or nighttime sleep duration among adolescents. * Methods*. Data were analyzed from 1,592 adolescents who completed an internet-delivered survey as part of the British Columbia Adolescent Substance Use Survey cohort study. Ordinal logistic and linear regression models were used to examine associations between frequency of SHS exposure in the past month and frequency of restless sleep and nighttime sleep duration, respectively. * Results*. SHS exposure was significantly positively associated with restless sleep and significantly negatively associated with sleep duration. In fully adjusted models, compared with students who reported never being exposed to SHS in the past month, students who reported a low, medium, or high frequency of SHS exposure were 1.53, 1.76, and 2.51 times as likely, respectively, to report more frequent restless sleep (OR = 1.53, 95% CI 1.08–2.16; OR = 1.76, 95% CI 1.22–2.53; OR = 2.51, 95% CI 1.59–3.98). With regard to sleep duration, as frequency of SHS exposure increased by one category, nighttime sleep duration during the week and weekend decreased by 4 minutes (*B* = −0.06, 95% CI = −0.01– − 0.11) and 6 minutes (*B* = −0.09, 95% CI = −0.03– − 0.14), respectively. * Conclusions*. This study suggests that frequency of SHS exposure has a significant dose-response relationship with restless sleep and sleep duration in adolescents.

## 1. Introduction 

Sleep is critical for adolescent health, development, and functioning. Inadequate sleep quantity and quality have been associated with poor school performance, mental health problems, poor sociability, behavioral problems, and the development of obesity and its accompanying comorbidities in adolescents [1–12]. Furthermore, sleep problems experienced during adolescence are associated with increased incidence of adulthood depression, anxiety, attention problems, and aggressive behaviors, thereby indicating a long-term effect of poor sleep on mental health [13]. Although individual variations exist with regard to sleep requirements, the Centers for Disease Control and Prevention and the National Sleep Foundation recommend that adolescents get an average of 8.5 to 9.5 hours of sleep during each 24-hour period [14]. Meanwhile, Canadian adolescents today are getting less sleep than in the past, and more than 25% experience some form of sleep problem [15, 16]. This reduction in sleep duration could be due to numerous factors, including but not limited to increased school demands (e.g., homework and early start times), social activities, sports, use of technology (e.g., television, computers, internet, cell phones, and video games), caffeine consumption, or tobacco smoke exposure.

Exposure to tobacco smoke in Canada remains prevalent, as an estimated 22% of adolescents in grades 5–12 are exposed daily or almost daily to secondhand smoke (SHS) in their home [17, 18]. While active cigarette smoking has been linked to sleep disturbances in previous studies, the impact of SHS exposure on sleep is less clear [19–24]. For example, several studies with children reported a positive association between SHS exposure and inadequate sleep [25, 26], while Davila et al. reported no significant association between SHS exposure and sleep disorders in a US population-based sample [23]. Among analyses that deemed tobacco smoke exposure a risk factor for inadequate sleep in children and/or adolescents, SHS exposure has been associated with greater sleep-onset latency, greater sleep disturbance, more frequent parasomnias, and more symptoms of sleep disordered breathing (e.g., snoring) [25–28]. Furthermore, children exposed to tobacco smoke either pre- or postnatally have poorer sleep quality and more symptoms of sleep-disordered breathing compared with children who were unexposed during these periods [25, 27].

While existing literature is suggestive of an association between tobacco smoke exposure and sleep difficulties in adolescents, it is limited in scope. Therefore, the primary objective of this study was to examine whether frequency of adolescents' SHS exposure was associated with frequency of restless sleep; the secondary objective was to assess the relationship between adolescents' SHS exposure and nighttime sleep duration. It was hypothesized that increased frequency of SHS exposure would be associated with increased frequency of restless sleep and reduced nighttime sleep duration.

## 2. Methods

### 2.1. Participants

Participants were 1,592 adolescents aged from 13 to 18 years who participated in an internet-based cohort study of adolescents in British Columbia, Canada (the BC Adolescent Substance Use Survey (BASUS)). BASUS recruited students from 48 public secondary schools using a variety of methods including class presentations from BASUS staff, in-school announcements, and posters and aimed to examine patterns of substance use including tobacco exposure [29]. The majority of students completed the survey online outside of school; however, some school administrators opted to have their students complete the survey in school computer labs during class. Eligibility criteria were ability to read and complete the internet-based survey in English and be 13 years of age or older. All participants provided informed consent, as well as written parental consent in schools requiring this. Upon completion of the survey, students received a $25 gift card. This study was approved by the University of British Columbia Behavioral Research Ethics Board. Data collection for this study occurred during Wave 5 of the BASUS survey (October through December of 2011). The survey, which was administered in the fall of 2011, was used to collect participant characteristics such as age, gender, ethnicity, subjective family income, smoking behavior, SHS exposure, and sleep quantity and quality.

### 2.2. Measurement of Exposure

We collected detailed questionnaire data regarding adolescents' exposure to SHS in the past month. Specifically, the following question was used to collect data on SHS exposure: “overall (excluding your own smoking) in the past month were you exposed to secondhand smoke?” with the following response options: (1) never, (2) at least once in the past month (low frequency), (3) at least once a week (medium frequency), and (4) every day or almost every day (high frequency). The questionnaire was also used to assess the existence of home cigarette smoking restrictions with the following yes/no question: “are there any restrictions against smoking cigarettes in your home?”

### 2.3. Outcomes of Interest

The primary outcome of interest was frequency of restless sleep, which was assessed with the statement, “during the past week my sleep was restless,” with the following response options: rarely or none of the time (<1 day); some or a little of the time (1-2 days); occasionally or a moderate amount of time (3-4 days); and most or all of the time (5–7 days). The secondary outcome of interest was nighttime sleep duration, which was assessed with the following questions. “What time do you usually go to bed on school nights?” “What time do you usually get up on school days?” “What time do you usually go to bed on the weekend?” “What time do you usually get up on the weekend?”

### 2.4. Covariates

Information on covariates collected in the questionnaire included age, gender, ethnicity, subjective socioeconomic status in terms of family income [30], and adolescents' smoking status. Ethnicity responses were coded as Caucasian, Aboriginal, Asian, or other. Subjective socioeconomic status was assessed by asking participants “how would you describe your household's financial situation (how much money your family has)?” Responses were made using a 7-point scale ranging from 1 to 7: (1) far above average, (2) quite a bit above average, (3) a little above average, (4) average, (5) a little below average, (6) quite a bit below average, and (7) far below average. Responses were collapsed to below average, average, or above average. Smoking status was assessed with the following yes/no question: “have you smoked cigarettes (the kind that come in a pack or roll-your-own)—any amount—at least once in the past 30 days?”

### 2.5. Statistical Analysis

First, we assessed frequencies, means and standard deviations of demographics, sleep characteristics, tobacco use, and SHS exposure variables. Multiple imputation was then used to create five datasets with missing values imputed on both the independent and dependent variables [31]. The imputed datasets were then used to examine the associations between frequency of SHS exposure and the sleep variables in two regression models: (1) unadjusted and (2) multivariate models adjusted for covariates previously shown to be related to adolescent sleep including age, gender, ethnicity, family income, and smoking status. Ordinal logistic regression was used in the restless sleep models and linear regression was used in the sleep duration models. An alpha level of *P* < 0.05 (2-tailed) was used to indicate statistical significance, and all statistical analyses were conducted using the Statistical Package for Social Sciences (SPSS) version 19.0.

## 3. Results

The characteristics of the sample are shown in Table 1. The mean age of the adolescents was 14.8 years, 59% were female, 60% were Caucasian, and 44% reported an above average family income. With regard to sleep characteristics, 50% reported rarely or never (<1 day per week) experiencing restless sleep, and the mean durations of sleep during the week (Monday to Friday) and weekend (Saturday to Sunday) were 8.6 and 9.8 hours, respectively. The majority (95%) of adolescents reported no smoking in the past 30 days; 85% reported having home smoking restrictions; and 43% reported SHS exposure at least once in the past month. The amount of missing data on each variable examined in this study varied from a maximum of 11% for subjective family income to less than 1% for age, gender, and smoking status.

### 3.1. Secondhand Smoke Exposure and Restless Sleep

**Figure 1:**
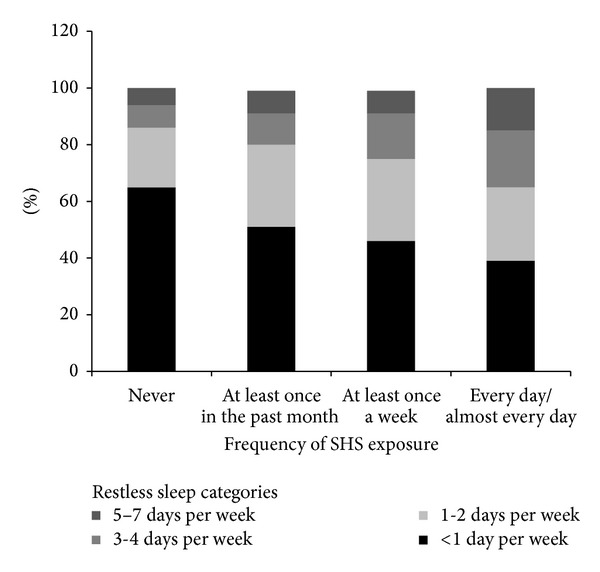
Frequency of restless sleep by frequency of secondhand smoke (SHS) exposure in the past month.

As displayed in Figure 1, there was a positive association suggestive of a dose-response relationship between frequency of SHS exposure and frequency of restless sleep. For example, among adolescents who reported never being exposed to SHS in the past month, 65% reported rarely or never (<1 day per week) experiencing restless sleep. In comparison, among adolescents who reported being exposed to SHS every day or almost every day, only 39% reported rarely or never (<1 day per week) experiencing restless sleep.

Frequency of overall SHS exposure in the past month was significantly associated with frequency of restless sleep in all unadjusted and adjusted ordinal logistic regression models.  Table 2 displays the odds ratios (OR) for the bivariate (unadjusted) and multivariate (adjusted) ordinal logistic regression models of restless sleep. Compared with students who reported never being exposed to SHS in the past month, students with a high frequency of SHS exposure had more than twice the odds (OR = 2.51, 95% CI = 1.59–3.98) of reporting more frequent restless sleep after controlling for age, gender, ethnicity, family income, and smoking status. The fully adjusted model also indicated that the odds of reporting more frequent restless sleep were 1.76 (OR = 1.76, 95% CI = 1.22–2.53) among students who reported a medium frequency of SHS exposure and 1.53 (OR = 1.53, 95% CI = 1.08–2.16) among students who reported a low frequency of SHS exposure compared with students who reported never being exposed to SHS in the past month.

### 3.2. Secondhand Smoke Exposure and Sleep Duration

**Figure 2:**
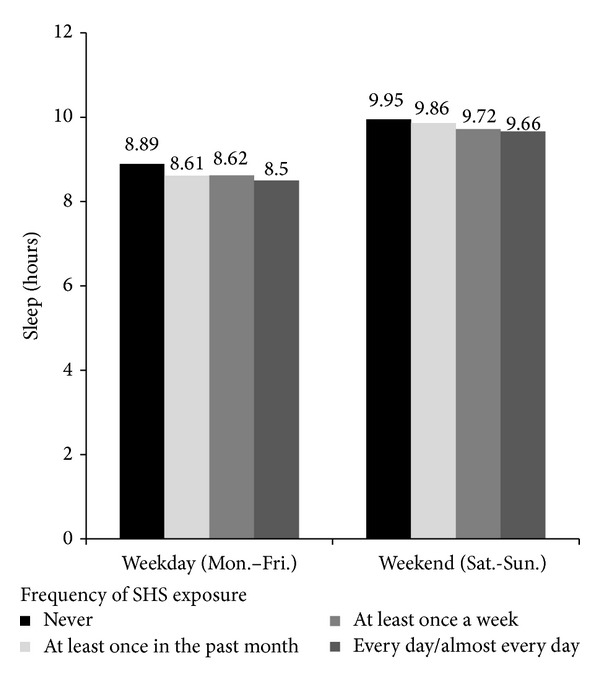
Sleep duration by secondhand smoke (SHS) exposure in the past month.

As displayed in Figure 2, adolescents who reported never being exposed to SHS slept an average of 8.9 and 10 hours during the week and on the weekend, respectively, while adolescents who reported a high frequency of SHS exposure reported an average of 8.5 and 9.7 hours of sleep during the week and on the weekend, respectively. In bivariate (unadjusted) and multivariate linear regression analyses, frequency of SHS exposure in the past month was significantly negatively associated with nighttime sleep duration during the week and weekend (Table 3). In the multivariate regression models that included potential covariates of adolescent sleep, we found that, as SHS exposure increased by one category, nighttime sleep duration during the week and weekend decreased by 4 minutes (*B* = −0.06, 95% CI = −0.01–−0.11) and 6 minutes (*B* = −0.09, 95% CI = −0.03–−0.14), respectively.

## 4. Discussion

The dose-response relationships between frequency of SHS exposure and frequency of restless sleep and sleep duration among adolescents suggested by these analyses add to the small but growing body of research pointing to SHS exposure as a risk factor for inadequate sleep in children and adolescents [25, 26, 32]. Our findings that indicate an association between SHS exposure and an increased likelihood of restless sleep among adolescents are consistent with a population-based cohort study in Sweden. Analysis of questionnaire data indicated that children aged three years who were exposed to SHS either pre- or postnatally had significantly poorer sleep compared with children of nonsmoking parents [25]. Also in line with our analyses are those of Yolton et al., who reported that increased SHS exposure was associated with greater sleep problems including disturbances, longer delays in sleep onset, sleep disordered breathing, and parasomnias in children [26]. Similar findings have been reported among adults. For example, Sabanayagam and Shankar reported that nonsmoking adults aged ≥20 years with SHS exposure had increased odds for insufficient sleep compared with unexposed nonsmokers [33]. Additionally, analyses by Ohida et al. suggested that nonsmoking pregnant women exposed to SHS reported more sleep difficulties including short sleep duration, insufficient sleep, and difficulty initiating sleep compared with those not exposed [34].

To our knowledge, this is the first study to examine the relationship between frequency of SHS exposure and nighttime sleep duration in a population-based sample of adolescents. Our findings, which show that adolescents' SHS exposure was significantly associated with reduced sleep duration, despite the relatively small effect, are in line with those of Nakata et al. who conducted the only known study that assessed SHS exposure and sleep duration among adults [35]. Data analyzed by Nakata et al. indicated that SHS exposure at work was associated with short sleep durations in adults [35].

Possible mechanisms underlying the present findings include the nicotine stimulating effect, nicotine withdrawal during sleep, changes in pulmonary function, and snoring-associated arousals [19, 26, 27, 32, 36–39]. SHS exposure may irritate the upper airway, exacerbating respiratory symptoms (e.g., sleep-disordered breathing), which in turn may lead to disturbed or restless sleep [26, 28]. Nonetheless, the precise mechanism(s) through which SHS exposure may impact adolescents' sleep remains uncertain. Additional studies on adolescents are warranted to confirm findings related to the associations between SHS exposure, restless sleep, and sleep duration, as well as examine underlying mechanisms.

## 5. Limitations

Several limitations should be mentioned. First, measures of SHS exposure, smoking status, and sleep quality and duration were based on self-report, which could have resulted in misclassification bias [35, 40]. Although self-reported sleep duration may have been overestimated, data from a study on adolescents indicated moderate correlations between self-reported usual weeknight sleep hours and actigraphy (Pearson's *r* = 0.53) [41]. Also, data from a study that compared adolescent-reported exposure to SHS with urinary cotinine levels indicated adequate exposure reporting, with a validity coefficient of *r* = 0.53 [42]. The inherent nature of the cross-sectional design does not permit inferences regarding directionality or causality of associations. Our sample included a small proportion (5%) of adolescents who reported smoking in the past month; however, we included smoking status in the adjusted models. Lastly, since data on physical activity, diet, body mass index, caffeine consumption, napping, snoring, and obstructive sleep apnea were unavailable, we were not able to adjust for these variables that may have impacted sleep.

## 6. Conclusions

Despite tobacco control efforts, a significant proportion of adolescents continue to be exposed to SHS. The results of this study indicate that frequency of SHS exposure has a significant dose-response relationship with restless sleep and sleep duration in adolescents. Among adolescents experiencing sleep-related problems, recommendations should include avoiding exposure to SHS. Further research using objective measures of SHS exposure (e.g., cotinine levels) and sleep (e.g., polysomnography) is needed to confirm findings in this study.

## Figures and Tables

**Table 1:** Characteristics of adolescents in a study on secondhand smoke (SHS) exposure and sleep (*N* = 1,592).

	Frequency of restless sleep^a^				Total^b^
	Rarely or none of the time (<1 day)	Some or a little of the time (1-2 days)	Occasionally or a moderate amount of the time (3-4 days)	Most or all of the time (5–7 days)	
*Demographics *					
					
Gender, *n* (%)					
Male	331 (45.5)	150 (37.3)	62 (32.5)	35 (26.9)	653 (41)
Female	396 (54.4)	252 (62.7)	129 (67.5)	95 (73.1)	931 (59)

Age, mean (SD)					
Years	14.8 (0.7)	14.8 (0.6)	14.9 (0.7)	14.9 (0.7)	14.8 (0.7)

Ethnicity, *n* (%)					
Caucasian	386 (55.7)	257 (66.6)	118 (64.5)	79 (64.2)	868 (60)
Aboriginal	22 (3.2)	12 (3.1)	9 (4.9)	6 (4.9)	55 (4)
Asian	250 (36.1)	96 (24.9)	50 (27.3)	33 (26.8)	456 (31)
Other	35 (5.1)	21 (5.4)	6 (3.3)	5 (4.1)	75 (5)

Family income, *n* (%)					
Below average	121 (17.9)	83 (22.3)	54 (29.5)	35 (29.4)	305 (22)
Average	236 (35.0)	132 (35.4)	62 (33.9)	39 (32.8)	492 (35)
Above average	318 (47.1)	158 (42.4)	67 (36.6)	45 (37.8)	615 (44)

Weekday nighttime sleep, mean (SD)					
Hours	8.7 (1.0)	8.7 (0.9)	8.5 (1.1)	8.2 (1.1)	8.6 (1.0)

Weekend nighttime sleep, mean (SD)					
Hours	9.8 (1.0)	9.9 (1.0)	9.6 (1.1)	9.7 (1.1)	9.8 (1.0)

*Adolescents' tobacco use *					
					
Smoked in the past 30 days, *n* (%)					
Yes	28 (3.9)	13 (3.2)	15 (7.7)	10 (7.7)	76 (5)

*SHS exposure *					
					
Overall SHS exposure in the past month, *n* (%)					
Never	133 (18.5)	44 (11.1)	17 (8.8)	12 (9.4)	236 (15)
At least once in the past month	321 (44.6)	184 (46.2)	72 (37.3)	52 (40.6)	661 (43)
At least once a week	187 (26.0)	118 (29.6)	64 (33.2)	34 (26.6)	423 (28)
Every day or almost every day	78 (10.8)	52 (13.1)	40 (20.7)	30 (23.4)	215 (14)

^a^These columns display data from participants who answered both the restless sleep and the specific demographic, tobacco use, and/or SHS exposure questions.

^b^This column displays data from participants who answered the specific demographic, tobacco use, and/or SHS exposure questions regardless of whether they answered the restless sleep question.

**Table 2:** Odds ratios (OR) for reporting more frequent restless sleep by frequency of SHS exposure among a sample of adolescents (*N* = 1,466).

Overall SHS exposure in the past month (versus never)^c^	Restless sleep^a^	
	Unadjusted OR (95% CI)	Full model^b^ OR (95% CI)
Never (reference)	—	—
At least once in the past month (low)	1.69^††^ (1.19–2.40)	1.53* (1.08–2.16)
At least once a week (medium)	2.01^††^ (1.39–2.89)	1.76^†^ (1.22–2.53)
Every day or almost every day (high)	3.05^††^ (2.00–4.67)	2.51^††^ (1.59–3.98)

CI: confidence interval.

^a^Ordered categories for increasing amounts of restless sleep are rarely or none of the time (<1 day per week), some or a little of the time (1-2 days per week), occasionally or a moderate amount of the time (3-4 days per week), and most or all of the time (5–7 days per week).

^b^Adjusted for age, gender, ethnicity, subjective family income, and smoking status (smoked in the past 30 days).

^c^Never was used as reference group.

Based on the test of parallel lines, the proportional odds assumption was held for all models.

*P < 0.05; ^†^ *P* < 0.01; ^††^ *P* ≤ 0.001.

**Table 3:** Associations between frequency of SHS exposure and sleep duration in a sample of adolescents (*N* = 1,466).

Overall SHS exposure in the past month	Nighttime sleep duration (hours) (Monday–Friday)	Nighttime sleep duration (hours) (Saturday-Sunday)
	B (95% CI)	B (95% CI)
Unadjusted	−0.09^††^ (−0.04–−0.14)	−0.09^††^ (−0.04–−0.14)
Full model^a^	−0.06* (−0.01–−0.11)	−0.09^††^ (−0.03–−0.14)

CI: confidence interval.

^a^Adjusted for age, gender, ethnicity, family income, and smoking status (smoked in the past 30 days).

*P < 0.05; ^†^ *P* < 0.01; ^††^ *P* ≤ 0.001.

## Abbreviations

BASUS: The BC Adolescent Substance Use Survey
CI: Confidence interval
OR: Odds ratio
SHS: Secondhand smoke.
